# Prenatal Exposure to Fenugreek Impairs Sensorimotor Development and the Operation of Spinal Cord Networks in Mice

**DOI:** 10.1371/journal.pone.0080013

**Published:** 2013-11-05

**Authors:** Loubna Khalki, Saadia Ba M’hamed, Zahra Sokar, Mohamed Bennis, Laurent Vinay, Hélène Bras, Jean-Charles Viemari

**Affiliations:** 1 Laboratoire Pharmacologie, Neurobiologie et Comportement, Centre National de la Recherche Scientifiques et Techniques (URAC 37), Cadi Ayyad Université, Marrakech, Maroc; 2 Institut de Neurosciences de la Timone, P3M Team, CNRS, Aix Marseille Université, Marseille, France; University of Edinburgh, United Kingdom

## Abstract

Fenugreek is a medicinal plant whose seeds are widely used in traditional medicine, mainly for its laxative, galactagogue and antidiabetic effects. However, consumption of fenugreek seeds during pregnancy has been associated with a range of congenital malformations, including hydrocephalus, anencephaly and spina bifida in humans. The present study was conducted to evaluate the effects of prenatal treatment of fenugreek seeds on the development of sensorimotor functions from birth to young adults. Pregnant mice were treated by gavage with 1g/kg/day of lyophilized fenugreek seeds aqueous extract (FSAE) or distilled water during the gestational period. Behavioral tests revealed in prenatally treated mice a significant delay in righting, cliff avoidance, negative geotaxis responses and the swimming development. In addition, extracellular recording of motor output in spinal cord isolated from neonatal mice showed that the frequency of spontaneous activity and fictive locomotion was reduced in FSAE-exposed mice. On the other hand, the cross-correlation coefficient in control mice was significantly more negative than in treated animals indicating that alternating patterns are deteriorated in FSAE-treated animals. At advanced age, prenatally treated mice displayed altered locomotor coordination in the rotarod test and also changes in static and dynamic parameters assessed by the CatWalk automated gait analysis system. We conclude that FSAE impairs sensorimotor and coordination functions not only in neonates but also in adult mice. Moreover, spinal neuronal networks are less excitable in prenatally FSAE-exposed mice suggesting that modifications within the central nervous system are responsible, at least in part, for the motor impairments.

## Introduction

Fenugreek (*Trigonella foenum graecum*) is one of the oldest traditional medicinal plants, cultivated in India, the Mediterranean region, North Africa and Yemen (Kassem et al., 2006). Fenugreek seeds are commonly used worldwide for its laxative and galactagogue properties but also to stimulate appetite and prevent stomach ache [1]. More recently, immunostimulatory, antidiabetic, antihypertensive and cholesterol-lowering potentials have been described [2-4]. However, consumption of fenugreek seeds during pregnancy has been associated with congenital malformations, neonatal and birth defects [5,6]. Oral treatment with the fenugreek seeds aqueous extract (FSAE) also causes reproductive and developmental toxicity [7-9]. Furthermore, we previously demonstrated that prenatal exposure to fenugreek seeds produced neurobehavioral alteration at P21 including decreased locomotor activity, impaired motor coordination and spatial short term memory [10]. Because of the widespread use of fenugreek seeds for its therapeutic actions during pregnancy, it is important to study the effects of FSAE on the development of neuronal networks such as the spinal locomotor network. Our aim was to investigate short and long term effects of FSAE exposure during gestation on locomotor functions, by using both *in vitro* and *in vivo* experimental paradigms. During the first postnatal week, spontaneous locomotor activity consists of crawling movements [11]. During this period, the hindlimbs remain passive. These movements are gradually replaced by crawling with all four limbs and around postnatal day 10, by walking on all fours with the belly free from the floor [12,13]. In spinal cord, a spontaneous bursting activity can be recorded *in vitro* from ventral roots during the perinatal period [14-16]. *In vitro*, a fictive locomotor pattern can also be elicited following bath application of excitatory amino acid agonists and monoamines such as serotonin (5HT) [17] or electrical stimulation of some brainstem areas [18-21]. At early postnatal days (P0-P3), we investigated whether spontaneous and locomotor-like activities were altered after prenatal exposure to FSAE. Then, at a later developmental period (P5-P12) we tested the pup‘s reflexes and swimming performance as indicators of the sensorimotor development. Finally, we assessed static and dynamic changes in juvenile (P21) and young adults (P41) using the CatWalk analysis. 

## Material and Methods

### Ethics statement

The experimental procedures conformed to the guidelines of the University of Marrakech and the European (Council Directive 86/6009/EEC) and French regulations (Ministry for Agriculture and Fisheries, Division of Animal Rights). Experimental procedures were approved by the Institut de Neuroscience de la Timone Ethics Committee registered at the National Commission of animal experimentation (autorized No. 71). Efforts were made to minimize the number of animals used. Adequate measures were taken to minimize pain and animal discomfort. 

### Preparation of extract

*Trigonella foenum-graecum* seeds were collected in the area of Settat, Morocco. The plant material was identified in the Department of Biology, Faculty of Sciences Semlalia, Cadi Ayyad University. A voucher specimen (n = 5511) was deposited at the herbarium of the mentioned faculty. The aqueous extract of fenugreek (*Trigonella foenum-graecum*) was obtained by agitating powder obtained from seeds in distilled water (1g/20 ml distilled water). The extract obtained was centrifuged and the supernatant was lyophilized (final yield of 20%) and stored at -20°C until further use.

### Animals and treatment

Swiss CD1 mice (25–30 g) were housed in groups of 4-5 with a 12 h light ⁄ dark cycle. Food and water were available *ad libitum* in the home cages. Males were housed overnight with females in proestrus stage (1: 2), and the females certified to be pregnant were divided into two groups of 17 animals. The first group used as controls received distilled water, while the second group was treated with aqueous extract of fenugreek (*Trigonella foenum-graecum*). Mice were treated by gavage with 1g/kg/day of lyophilized FSAE during the gestational period [9,10]. Doses up to 100 g/day, are reported to be taken by pregnant women [5]. The American Federal Drug Administration (FDA) Guidance for Industry (2005) provided an equation that enables to identify the human equivalent dose (HED) from animal data [22]. Translation of our data from mice weighting 25g to humans with a weight of 70 kg is given by the following equation:

HED = Animal Dose (mg/kg) x (animal wt/human wt in kg)^0.33^

 = (1000 mg/kg) x (0.025 kg/70kg)^0.33^

 = 72.85 mg/kg = 5.1 g/70 kg

This daily dose of 5.1 g appears to be on the very low side of human consumption.

### Evaluation of short term effects of prenatal exposure to fenugreek

We investigated the righting reflex and the cliff avoidance during the first postnatal week. and the geotaxis response at P10 and P12 [23].

***Surfacerightingreflex*** *(P5, P7 and P9*): this test evaluates motor function and coordination. Each pup was placed on its back on a flat surface and released. The time required to get back on all four paws was measured. The maximum time allotted to each trial was 30 seconds. ***Cliffavoidance*** (*P6*): the pup was placed on a table edge with the forepaws and nose over the edge. The time taken to make a U- turn was noted. Each pup was tested once. The maximum time allowed per trial was 60 seconds. ***Negativegeotaxis*** (*P10 and P12*): coordination can be examined using the negative geotaxis test, an automatic, stimulus-bound orientation movement which provides informations about vestibular and/or proprioceptive functions. The time taken to make a U-turn from a head-down position on a 35-degrees inclined plywood surface was measured. This test has been performed after P8 because it requires crawling movements that appear at the end of the first postnatal week [23].

***Swimmingdevelopment***: as newborn mice are able to swim straight at P8 [23], we started to analyse swimming at this age and repeated the same analysis at P10 and P12. Each mouse was placed in a water tank (28°C) for 5–10s. The direction, angle in the water (head position) and limb involvement were observed and scored. Direction was scored as follows: sinking: 0; floating : 1; circling : 2; swimming straight or nearly straight : 3. Angle scores consisted of head submerged : 0; nose at the surface : 1; nose and top of head at or above the surface but ears still below the surface: 2; ears half way above the surface : 3; ears completely above the surface : 4. The involvement of limbs was evaluated by the following scores : no paddling, 0; paddling with all four limbs : 1; paddling with only the hind limbs and the forelimbs remaining motionless : 2. The experimenters conducting the behavioral tests were blinded to the animal condition.

### Electrophysiological recordings

Mice (post-natal day P0- P 3) were deeply anesthetized by hypothermia. After decapitation, the lumbar spinal cord was dissected in an artiﬁcial cerebrospinal ﬂuid of the following composition (in mM): 128 NaCl, 4 KCl, 1.5 CaCl2, 1 MgSO4, 0.5 NaH2PO4, 21 NaHCO3 and 30 glucose; oxygenated with 95% O2 and 5% CO2, pH 7.4. The spinal cord and roots were removed from sacral segments up to T8-T10. The preparation was pinned down, ventral side up, in the recording chamber, and continuously perfused with the same saline solution as the one used for dissection. The temperature was set to 28–30°C. Spontaneous and locomotor-like activities were recorded from lumbar ventral roots (left ⁄ right L2 and L5) by means of glass suction electrodes connected to an AC-coupled amplifier (bandwidth: 70 Hz to 3 kHz). Fictive locomotion was elicited by a bath application of N-methyl-DL-aspartic acid (NMA) ⁄ 5-hydroxytryptamine creatinine sulfate (5-HT) for 30min (NMA, 10 µM; 5-HT, 10 µM). All compounds used were purchased from Sigma. Electrophysiological data were acquired through a Digidata 1440A interface using the clampex 10 software (Molecular Devices, Sunnyvale, CA, USA).

Data analysis consisted of rectifying and integrating (time constant 50 ms) the raw extracellular recordings from ventral roots. The phase relationships were evaluated on 10 successive 60-s recordings by means of cross-correlation analysis. The locomotor-related coordinations were assessed by the cross-correlation coefﬁcient at zero phase lag (center of the cross-correlogram). The mean correlation coefﬁcient (R) is the average of r values collected from all animals a given experimental condition. Bursts were automatically detected with clampﬁt 10.0 software (Molecular Devices). After setting a Y-threshold value in the recording, the program detects events above this value”. We then used the “analysis event” function and then the “burst analysis” tool that calculates the mean duration and the frequency of bursts [16].

### Evaluation of long term effects of prenatal exposure to fenugreek

Motor ability and coordination were evaluated using a Rotarod apparatus (Orchid Scientifics) for mice (25 cm length, 4 cm diameter). The ability of five-weeks-old mice to keep their balance on a rotating bar (10 turn/min) was tested during 3-min trials and the fall latency was measured. Mice underwent two trials separated by a 15 minutes interval. 

The CatWalk system (CatWalk XT 9.1, Noldus Information Technology bv, Wageningen, The Netherlands) was used to evaluate gait parameters. All the characteristics of the system have been described previously [24-26]. The test was performed on ten males (five controls and five FSAE prenatally exposed mice) at P21 and P41. Mice freely ran accross a glass walkway located in a dark corridor (width 20 cm, length 150 cm). A combination of green LED lighting of the glass plate and red light illumination of the ceiling enabled to visualize the paw prints and the body contour of the animal which were recorded using a high-speed digital camera (10 frames/s) positioned beneath. Only runs with a maximum speed variation of 30% were kept for analysis. At least three runs were collected for each session. Gait parameters can be classified into three categories: 1) static paw parameters: such as intensity of the paw print, print area, base-of-support, stride length, paw angle and relative paw position; 2) dynamic paw parameters: step cycle, stance and swing durations; 3) interlimb coordination such as step patterns, regularity index (RI) and the phase lags. A full description of the locomotor parameters is provided by Hamers [26].

### Statistical analysis

Data were analyzed using Prism 4 software (GraphPad) and are presented as mean and standard errors. Differences between groups were tested by ANOVA 2 variance and the Mann Whitney non parametric test. Chi- square test was used to analyze the step patterns in the gait analysis. Linear regression was also used to assess whether the time required for the righting reflex was correlated with postnatal age. All values are presented as mean ± sem. The level of significance was p ≤ 0.05.

## Results

Short (P0-P12) and long term effects (P21-P42) of prenatal FSAE exposure on the locomotor activity were investigated using electrophysiological recordings, behavioral tests and gait analysis.

### Prenatal exposure to FSAE affected the sensorimotor development

**Figure 1:**
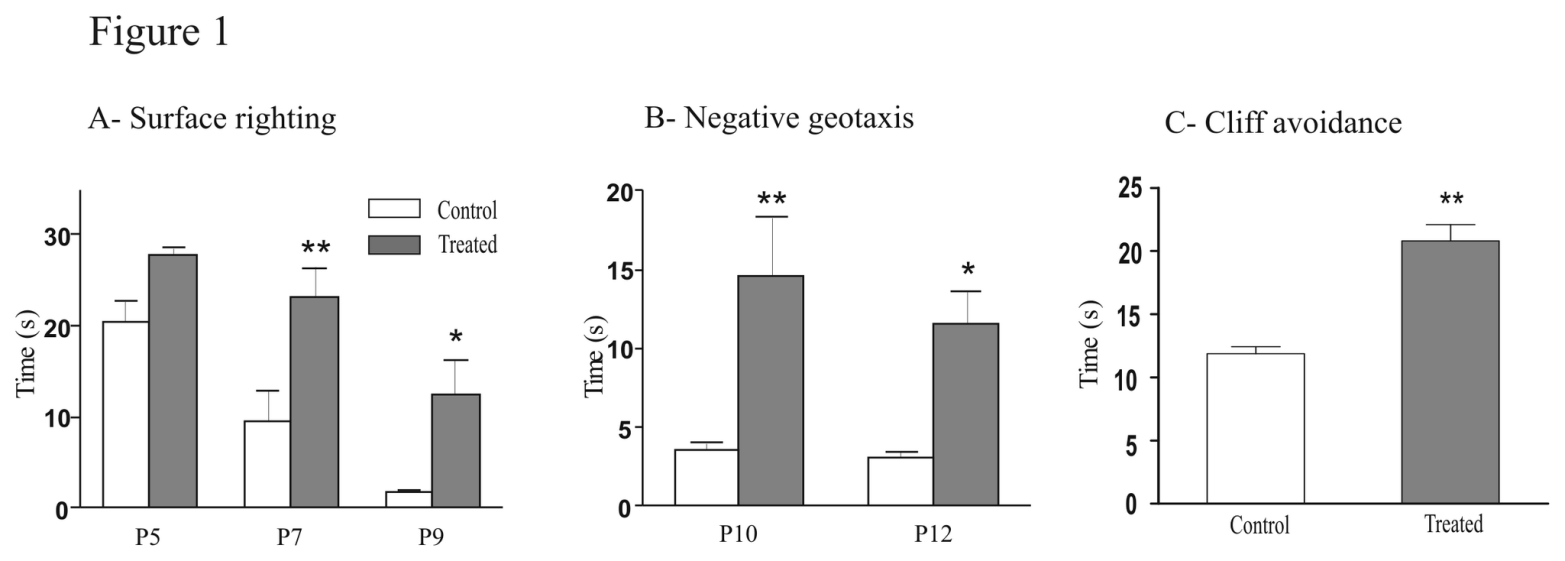
Treatment of pregnant mice with fenugreek seeds extract delayed sensori motor performances. (A) The righting response was significantly longer in FSAE exposed pups compared with control at P7 and P9. (B) When placed on a 35° angle slope, the time for the treated mice to turn around to orient their body with the head upward significantly increased at P10 and P12. (C) The time that the treated mice take to turn and crawl away from the cliff drop is significantly increased. (n = 6). *P < 0.05, **P < 0.01.

**Figure 2:**
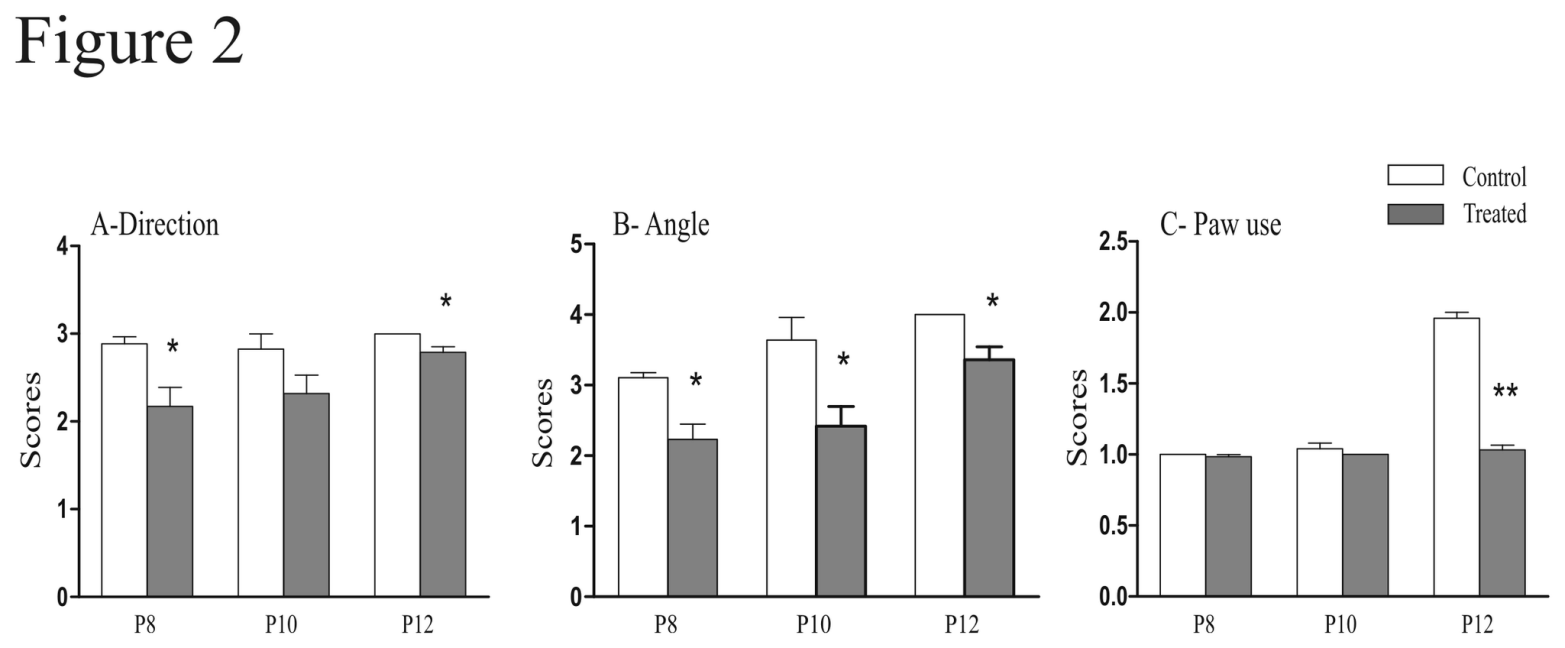
Fenugreek exposure affected swimming performances. We scored the direction of swimming in A. (0; floating, 1; circling, 2; swimming straight or nearly straight, 3), the angle of the head in B (head submerged, 0; nose at the surface, 1; nose and top of head at or above the surface but ears still below the surface, 2; ears half way above the surface, 3; ears completely above the surface,4) and the use of paws in C (0; paddling with all four limbs, 1; paddling with only the hind limbs and the forelimbs remaining motionless, 2). Control mice had a higher performance compared with treated mice, (n = 6). *P < 0.05, **P < 0.01.

We tested the influence of FSAE exposure on sensorimotor tests performed during the postnatal period to evaluate the proper development of the sensorimotor capacities. First, we studied the surface righting reflex that requires the integrity of muscular and motor functions as well as labyrinthine ones. The time required for the surface righting response significantly decreased with age in controls (Spearman Test, P = 0.0049) whereas it did not significantly change in FSAE treated mice (Spearman Test, P = 0.15). Moreover, the righting response was significantly longer in FSAE exposed pups compared with controls at P7 (23.3 ± 3.1 s vs 9.4 ± 3.4 s, (t = 3.7; P< 0.01) and P9 (12.4 ± 3.7 s vs 1.7 ± 0.3 s, t = 2.9, P < 0.05; Figure 1A). Second, we evaluated the geotaxis reflex. When placed on a 35° angle slope, most control mice turned around to orient their body with the head upward within 4s (Figure 1B). This negative geotaxis response was much slower in treated mice as reflected by a ~300% increase in time at both ages tested [P10 (t = 3.9; P < 0.01) and P12 (t = 3.1; P < 0.05)]. Third, we performed the cliff avoidance test: the time taken by treated animals to turn and crawl away from the cliff drop was significantly increased compared with controls (20.8 ± 2.9 s vs 11.9 ± 1.2 s in control; P < 0.01; Figure 1C). We also analyzed the swimming development, with regard to the swimming direction, the treated group had a lower score than the controls at P8 (t = 9; P <0.001), P10 (t= 8.6; P < 0.001) and P12 (t= 9.3; P <0.001) (Figure 2A). There were also significant differences for the swimming angle [P8 (t = 2.9; P <0.05); P10 (t= 4.1; P <0.01)]. However, at P8 and P12, treated mice used all four limbs as frequently as controls. Then at P12, the score became significantly lower in treated animals (t = 23.8; P <0.001) (Figure 2C).

### FSAE decreased the excitability of spinal networks

**Figure 3:**
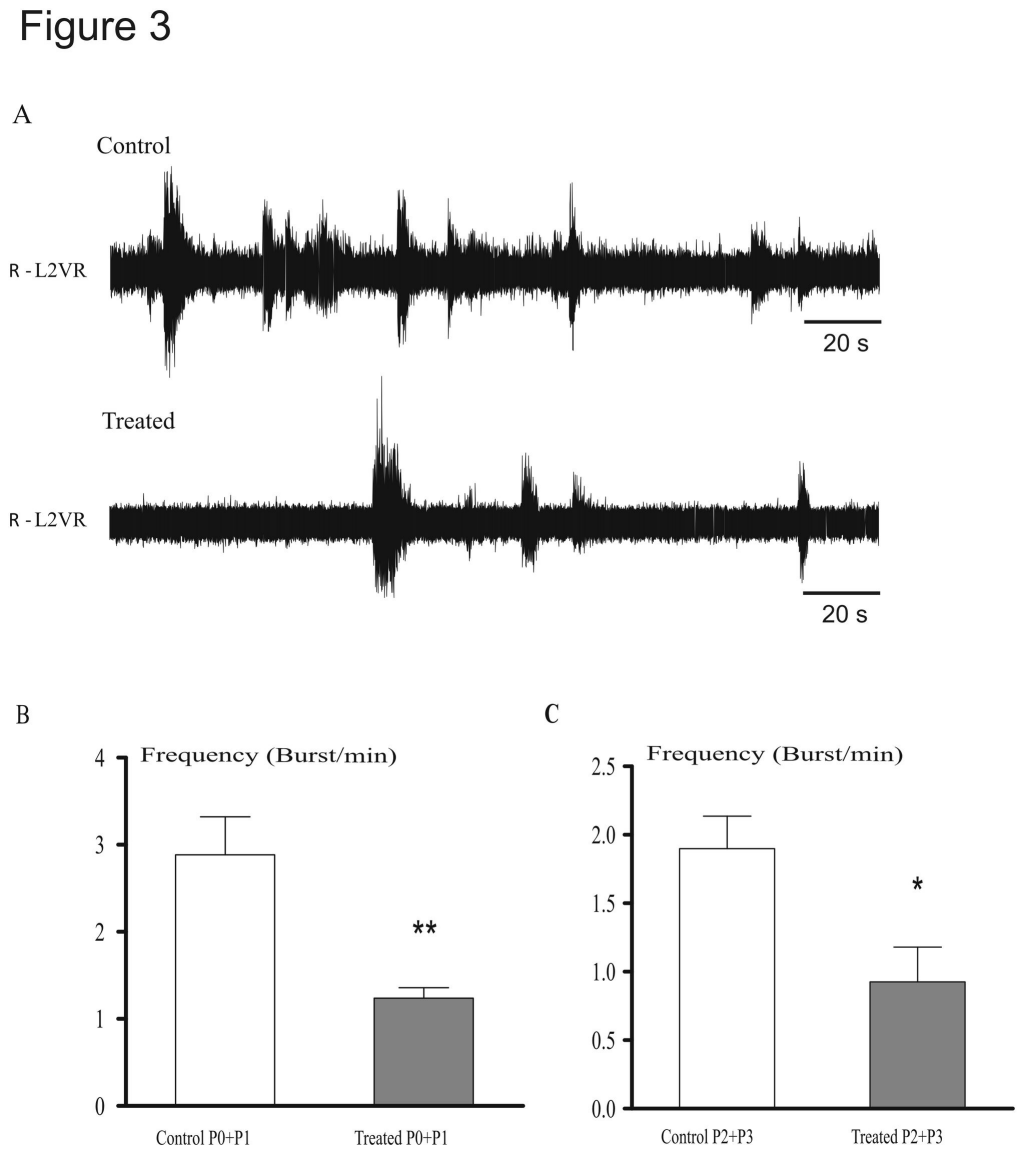
Fenugreek exposure reduced the frequency of spontaneous bursts. (A) Spontaneous bursts of neuronal activity were recorded from L2 lumbar ventral roots in control (upper trace) and treated (lower trace) pups. (B) The frequency of spontaneous bursts was significantly lower in treated compared with control mice at different ages (P0- P1 and P2-P3). *P < 0.05, **P < 0.01.

We then examined whether spontaneous activity in the spinal cord was altered in prenatally treated mice. In control mice, recordings from L2 ventral roots (Figure 3A) revealed bursts of spontaneous activity at a frequency of 2.88 ± 0.43 at P0-P1 and 1.9 ± 0.24 bursts/min at P2-P3, respectively. This frequency was significantly reduced in treated mice (1.23 ± 0.12, U = 5, P = 0.009 and 0.92 ± 0.26, U = 3, P = 0.04 at P0-P1 and P2-P3, respectively; Figure 3B, 3C). 

**Figure 4:**
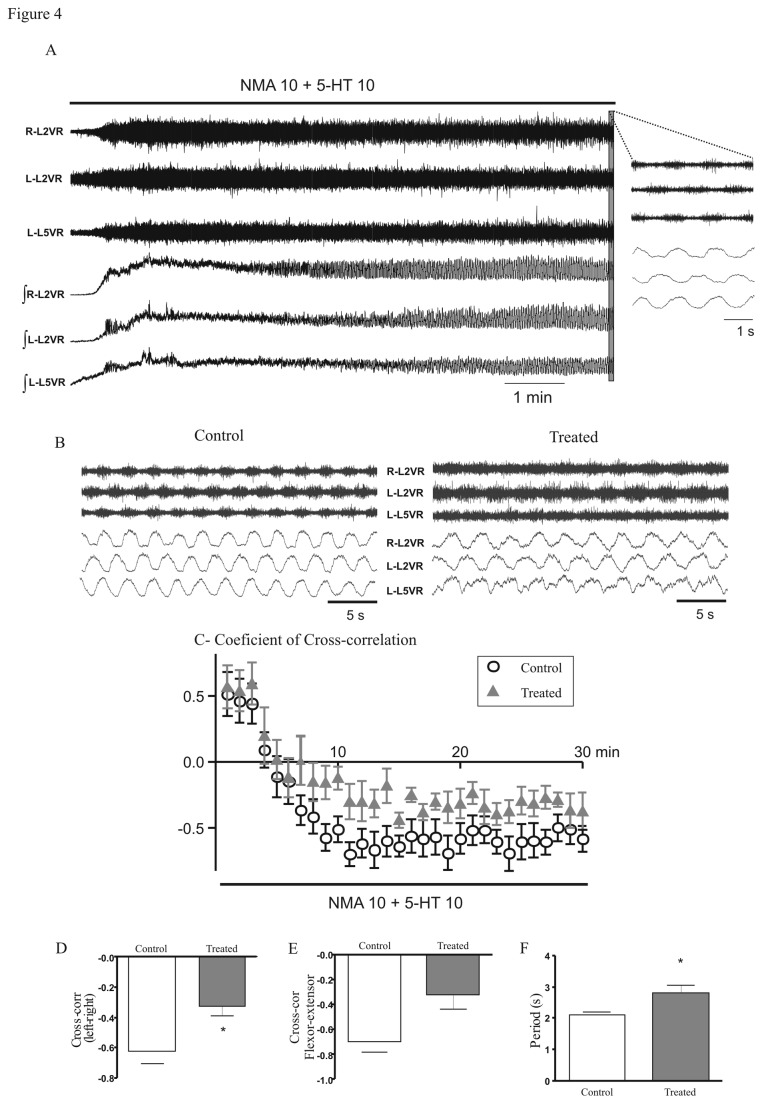
Fenugreek exposure decreased the frequency and the alternations of the locomotor like activity. (A) NMA ⁄ 5-HT induced locomotor like activity in control. The top and bottom traces represent the row and the integrated signals, respectively. Traces on the right represent the enlargement of the grey rectangle, the trace shows the alternation of motor bursts 10 minutes after the drugs application. (B) Fictive locomotion induced in control and treated mice. An alternating pattern was observed in both groups. (C) The graph shows the evolution of the left-right alternation during the 30 min of the NMA/5-HT exposure. Triangles and circles represent the evolution of the cross-correlation coefficient for treated and control mice, respectively. When this coefficient is positive the traces are in phase and when the coefficient is negative the traces are out of phase and the pattern is alternating. (D) The cross-correlation coefficient calculated for the opposite ventral roots (L2) calculated between the 20-30 minutes time window was more negative in control than in treated mice (Control: n = 7; treated: n = 7). (E) There was a non-significant trend towards less negative values for the cross-correlation coefficient calculated from the L2 (extensor) and L5 (flexor) ventral roots in prenatally treated mice compared with controls (Control: n = 4; treated: n = 3). (F) The period of locomotor-like activity is shorter in control compared with prenatally treated mice. Periods were compared during the 20-30 minutes time window. *P < 0.05.

Application of NMA and 5-HT on spinal cord preparations induced ﬁctive locomotion characterized by left–right and ipsilateral flexor–extensor alternations. After an initial phase of induction (Figure 4A, 0-10min), the locomotor pattern was characterized by negative values of the cross-correlation coefficient that reached the most negative values after 20 minutes of NMA/5-HT application. During this period (20-30 min), recordings from L2 and L5 ventral roots showed alternated rhythmic discharges in control and prenatally treated mice (Figure 4B). The cross correlation coefficient in treated mice was significantly less negative than in controls (Figure 4C, D, E). The locomotor period was also significantly longer in treated mice compared with controls (2.81 ± 0.25 s and 2.09 ± 0.11 s respectively, U = 7.5; P = 0.03; Figure 4F).

### Long term effects of prenatal exposure to FSAE on motor coordination and gait

Animals of both groups were regularly weighed along the developmental period. The FSAE treated mice had a significant lower weight at P21 and P28 (18.4 ± 1.14 g and 25.2 ± 1.09 g compared with 21.2 ± 0.8 g and 28 ± 1 g in control). However, the weight of treated and control was not siginificantly different at P34 and P41. We investigated the motor coordination by means of the rotarod test. Two trials were performed in each group. Performances of control mice on the rotarod were always better than those of FSAE treated mice. Prenatally exposed mice spent a significantly shorter time on the rotarod than the controls in each trial (11.4 ± 2.6 s vs 77.1 ± 11 s for the first trial, U = 1; P = 0.0002 and 36 ± 8.4 s vs 142.6 ± 14 s for the second, U = 2; P = 0.0002).

**Figure 5:**
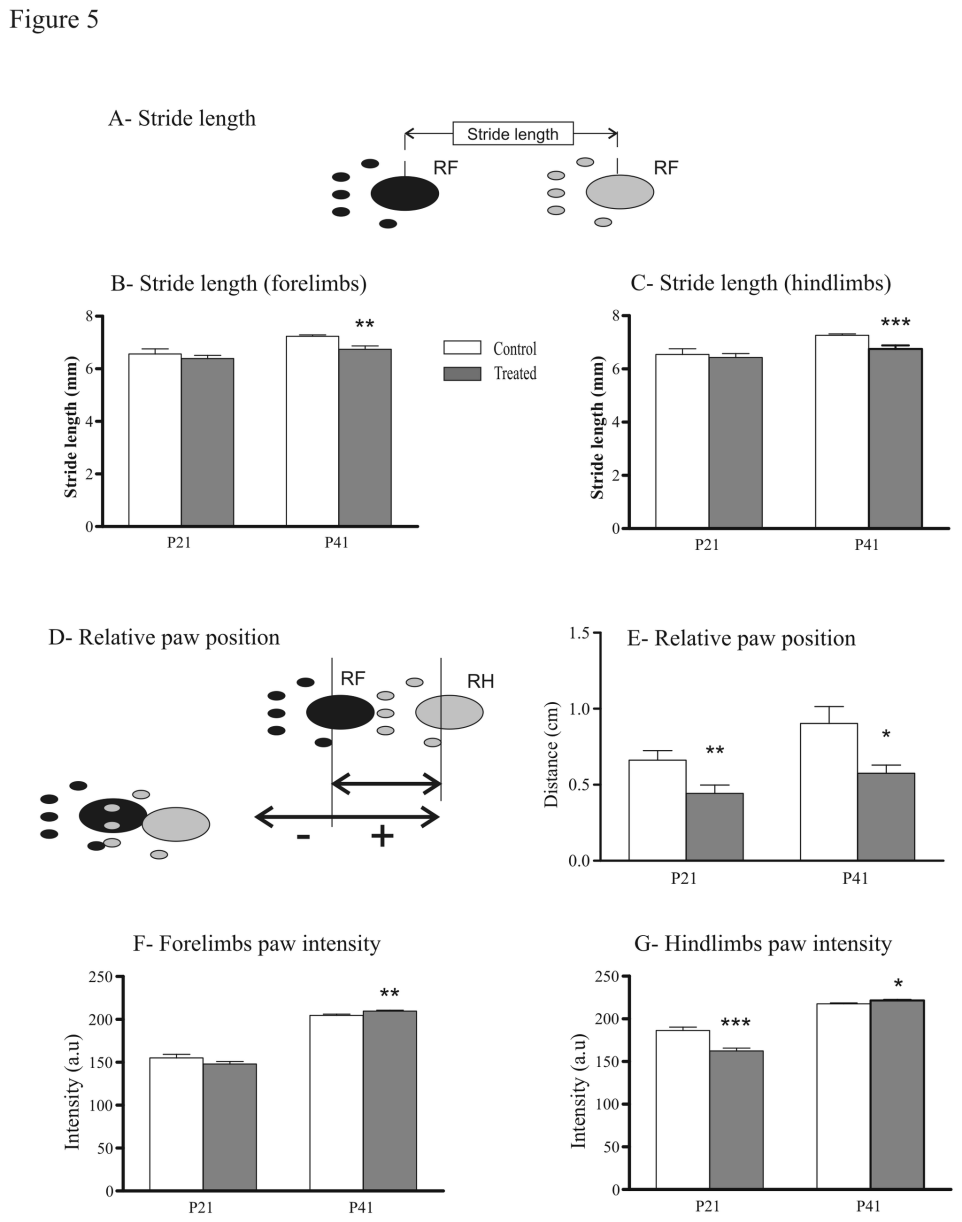
Effects of fenugreek exposure on gait spatial parameters. (A) Schematic of the stride length (distance between successive placements of the same paw). Stride length of forelimbs (B) and hindlimbs (C) was significantly shorter in prenatally treated mice when compared with controls. (D) Schematic of the paw positioning: position of the hindpaw compared with position of the previously placed ipsilateral forepaw. (E) The position of the hindpaw compared with the forepaw is closer in the treated mice at P21 and P41. (F) The forelimbs paw intensity (mean brightness of the pixels of the print) of treated mice was significantly stronger at P41. (G) The hindlimbs paw intensity of treated mice was significantly decreased at P21 and increased at P41. *P<0.05, **P<0.01, ***P<0.001.

We then analyzed the static gait parameters. The base-of-support (BOS) for both fore- and hindlimbs was identical in treated and control groups. We measured the stride lengths of forepaws and hindpaws, the distance between consecutive steps (Figure 5A). The stride lengths were not significantly different at P21. At P41, it was shorter in prenatally FSAE treated mice (6.75 ± 0.1mm vs 7.26 ± 0.05 mm, U = 6; P = 0.0003 for the hindpaws and 6.74 ± 0.1 mm vs 7.2 ± 0.05 mm for the forepaws, U = 10; P = 0.001; Figure 5B, 5C). Regarding the relative paw position (Figure 5D), control mice placed their hindpaws 7 mm and 10 mm ahead of the position of the ipsilateral forepaws at P21 at P41, respectively. In treated mice, this distance was smaller [0.4 cm at P21 (U = 12; P = 0.005) and 0.6 cm at P41 (U = 0; P = 0.007), Figure 5E). We then calculated the paw intensity, the mean intensity with which the paw is placed was computed over the whole stance period. At P21, treated mice hindpaws intensity is lower compared with controls (U = 51; P = 0.0005). However, at P41 the intensity increased and was significantly higher in both fore- (U = 13; P = 0.003) and hindlimbs (U = 14; P = 0.01) in treated mice compared with controls (Figure 5F,G). 

**Figure 6:**
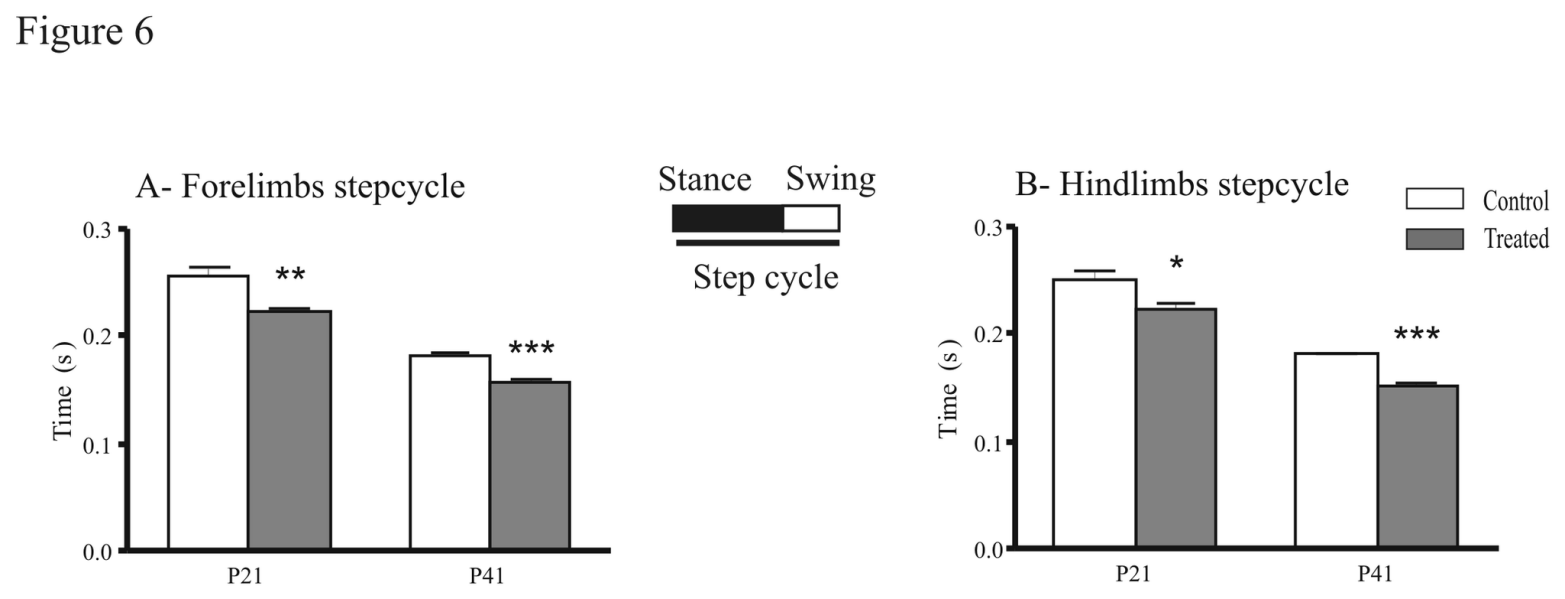
Effects of fenugreek on the step cycle duration. Schematic illustrating the step cycle composed of the stance and the swing phase. The step cycle duration (time in second between two consecutive contacts of the same paw) of treated mice is significantly shorter for the forelimbs (A) and the hindlimbs (B) at P21 and P41 compared with controls. *P<0.05, **P<0.01, ***P<0.001.

**Figure 7:**
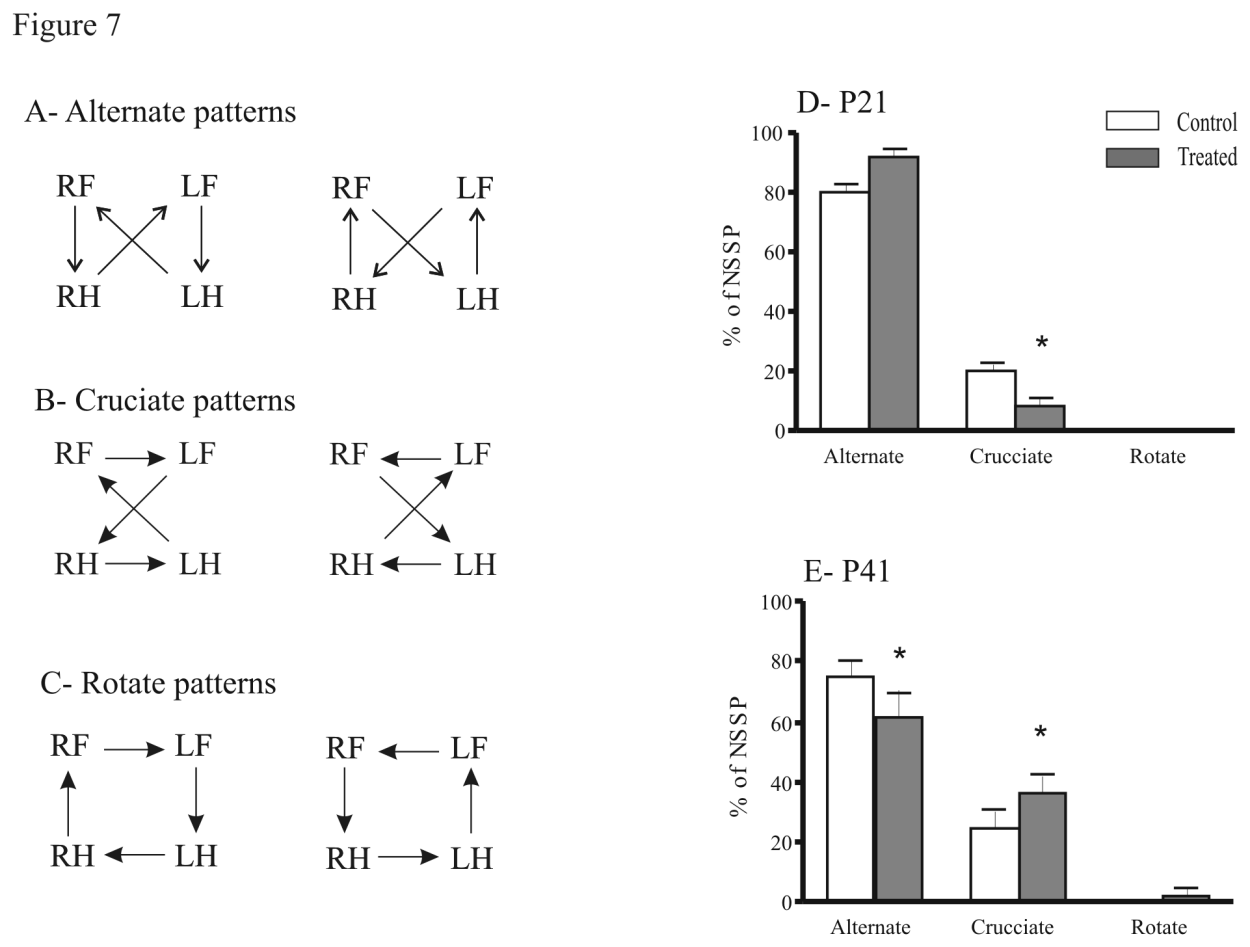
Effects of fenugreek exposure on the different locomotor patterns. (A-C) Schemes illustrating the alternate (A), cruciate (B), and rotate (C) patterns. Control and treated mice predominantly used the alternate patterns at P21 (D) and P41 (E). At P41 (E), the treated mice used more frequently the cruccciate and the rotate patterns than the controls. *P<0.05. Pattern adapted from Cheng et al. [24]. RF, right forepaw; LF, left forepaw; RH, right hindpaw; LH, left hindpaw.

Finally, we analyzed the dynamic gait parameters: the stance duration of treated mice was significantly shorter at P41 in both fore- (U = 20; P = 0.02) and hindlimbs (U = 20; P = 0.02) compared to control animals. The differences between control and treated groups in the step cycle duration were similar for the forelimbs and the hindlimbs (Figure 6). The stepcycle duration of treated mice was significantly shorter compared to the control group in forelimbs [P21 (U = 85; P = 0.015); P41 (U = 2; P = 0.0002); Figure 6A] and hindlimbs [P21 (U = 69; P = 0.003); P41 (U = 0; P < 0.0001); Figure 6B]. Regularity index (RI) indicates the regularity in which the animal uses normal step sequence pattern (NSSP), taking into account the order of paw placements [27]. The RI did not differ between the treated and the control groups (data not shown). Concerning regular step patterns, in control animals, only two step patterns were detected: the alternate pattern (Figure 7A), which is predominant at P21 (80 ± 2.8 %, Figure 7D), and P41 (75 ± 5.5 %, Figure 7E) and the crucciate pattern (Figure 7B). Prenatal exposure to FSAE led to a different use of step patterns compared to control mice. At P21, the crucciate pattern was significantly less represented in the treated group than in the control (8 ± 2.9 % vs 20 ± 2.8 %, respectively; Figure 7D; U = 2, P = 0.03). At P41, the alternate pattern was significantly reduced in treated mice (-33%), to the profit of the crucciate patterns which reached 36 ± 6.25 %. The use of rotate pattern (Figure 7C) was absent at P21 and remained so in adult mice (P41) in both treated and control groups (Figure 7E). In contrast to the RI and the six NSSP, the phase lags is a measure of the coordination with respect to timing of paw placement [27]. No significant differences between groups were noticed in the ipsilateral-, diagonal- and girdle-phase lags.

## Discussion

Fenugeek has been used since antiquity by Indian and North African populations for its laxative and galactagogue effects [1]. Today, fenugreek capsules are freely available, mainly as an appetite stimulant, but immunostimulatory, antidiabetic, antihypertensive and cholesterol-lowering properties have been put forward [2-4,28]. In view of the widespread use of fenugreek and the lack of medical monitoring, the safety of fenugreek seeds consumption during pregnancy turns out to be a public health concern. So far, there are no scientific reports regarding the dose of Fenugreek that can be at risk. However, human consumption can rise up to 100g/day. In this study we showed that in mice, a dose of 1g/kg/day that would correspond in humans to a dose as low as 5g/day (see Methods) may affect the development of spinal networks.

A clinical study reported a number of cases of malformations in new born whose mothers consumed fenugreek seeds during pregnancy [6]. Fenugreek seed was shown to have an antifertility effect on both male and female treated rats [7,8]. Previous results from our laboratory have demonstrated that the oral treatment of mice with FSAE induces reproductive and developmental toxicity [9] as well as neurobehavioral alteration at P21 [10]. Here, we investigated the short and long-term effects of exposure to FSAE on motor function in mice. We show that FSAE exposure decreases the frequency of both spontaneous and locomotor-like activity in vitro, delays sensorimotor development and impairs gait parameters in juvenile (P21) and young adult (P41) mice. Taken together, these data reveal long-lasting alterations in sensorimotor development and motor coordination after prenatal exposure to FSAE. 

### FSAE delayed pup’s sensorimotor development

Maturation of posture is a limiting factor with regard to locomotor performance in immature rodents [11,29]. As a result, swimming which removes postural constraints may be used as a criterion of locomotor network maturation [30]. Swimming is a good index of neuromuscular development and integration because of the number of neurological and muscular systems that need to be coordinated [31]. In our study, FSAE prenatally treated mice exhibited a different swimming pattern characterized by delayed performance in the direction of swimming which can be attributed to uncoordinated movement of the legs [32]. The mature swimming pattern takes place during the second post-natal week and is characterized by the use of the 2 legs [32]. FSAE-treated mice had a lower score which can be due to impaired development of the motor or neuromuscular systems.

Regarding the innate reflexes, we noticed that there was a deficit in the righting reflex, the negative geotaxis and the avoidance of fall. The first two tests enable to assess vestibular (inner ear) and / or proprioceptive related aspects [33]. Moreover, a lack of coordination in the righting reflex has been reported as one of the deficits that accompany the development of mouse cerebellum [34]. Fall avoidance enables to evaluate the maturity of sensorimotor functions [35]. We can conclude that the failure and / or delay in the achievement of these reflexes in our treated mice are probably due to impaired development of motor and vestibular systems. However, we cannot exclude that these deficits arise from defects in the motor control circuitry as discussed below. 

### FSAE decreased the frequency of spontaneous activity and fictive locomotion

Spontaneous activity plays a fundamental role during the development of neural networks [36]. Several studies provided evidences that modifications of neuronal activity during development alters, for instance, the neurotransmitter phenotype of spinal neurons [37,38] and induce changes in locomotor outputs [39]. Spontaneous activity is essential for the development of the functional architecture of neuronal networks [40,41] and for the incorporation of newly born neurons in the adult nervous system [42]. Spontaneous bursts of electrical activity of developing neurons are important not only for the refinement of neuronal connections but also for correct path finding [15]. Here, we investigated the effects of FSAE exposure on the excitability of the spinal network. Prenatally FSAE exposed P0-P3 pups exhibited a marked decrease in frequency of spontaneous activity compared with control mice. 

Changes in the frequency of the spontaneous activity may reflect modification of chloride homeostasis [16]. Early during development, chloride mediated neurotransmission is excitatory [43] so that the spontaneous activity is intense and consists of long-lasting bursts separated by periods of quiescent activity, due to the hyper excitable nature of networks. In the rodent ventral spinal horn, the switch from depolarizing to hyperpolarizing actions of GABA and glycine occurs during the first post natal week [44,45] and is promoted by the up-regulation of the expression of the potassium-chloride co-transporter KCC2 [46,47] with the maturation of the expression of GABA and glycine receptor-gated chloride channels [48]. The decreased excitability of the spinal network due to FSAE exposure during the prenatal period also leads to a slower frequency of fictive locomotion. We induced fictive locomotion by adding NMA and 5-HT which triggered stable alternating rhythms in controls. In FSAE exposed mice, left/right and flexor/extensor alternations were still observed but the cross-correlation coefficient was less negative. It has been shown that the frequency of the locomotor rhythm in vitro as well as the quality of the alternating pattern depends on the concentration of NMA which tunes the excitability of the network [16,49]. The slower frequency of fictive locomotion and the deteriorated alternating pattern after FSAE exposure during the prenatal period may therefore reflect a decreased excitability of the spinal networks and/or motoneurons. 

### FSAE Impaired Motor coordination

In quadrupeds, walking on a flat surface, which is the pre-requisite paradigm for Catwalk analysis, is coordinated by spinal neuronal networks that generate a basic motor pattern [50]. The generated pattern is characterized by a rhythmic alternation between left and right as well as flexor and extensor muscles of the limbs and probably only initiated and terminated by higher motor centers. The most common regular walking pattern [25-27], is the alternate pattern, constituting 80% to 95% of the total step cycle in intact rats and mice, respectively [24]. Our results also confirmed that mice preferentially used the alternate pattern and to a lesser extent the cruciate pattern. However, in FSAE treated mice, only 60% of step sequences presented an alternate pattern at P41, whereas the proportion of steps with a cruciate pattern significantly increased (36%). This change of step pattern might be related to alteration in the sequence of limb placement due to motor asymmetry [51]. The rotarod test, a classical neurologic test which evaluates fine motor function and coordination [52], showed that at P42, prenatally treated mice had a significantly shorter fall latency compared with control. FSAE treated mice were able to improve but they still had weaker motor coordination (by 80%) as previously reported at P22 [10]. These results suggest that FSAE exposure affected the motor coordination, in support of our *in vitro* experiments. 

#### FSAE exposure induced changes in gait parameters

In intact animals and humans, locomotor speed increases as a result of the shortening of the stance phase [53]. In our study, the mean locomotor speed of the control and treated groups were similar. However, FSAE exposure reduced the floor contact during walking (short stance duration) and the step cycle duration. There is a discrepancy between the effects of FSAE exposure on cycle duration between *in vivo* (shortening) and in vitro (lengthening) data. It is difficult to compare *in vitro* and *in vivo* conditions for at least three sets of reasons. 1/ Only the spinal cord was retained in vitro whereas all modulatory influences –including monoamines- from supraspinal centers and sensory inputs were available *in vivo*. 2/ The activity of spinal cord locomotor networks was induced pharmacologically in vitro whereas locomotion occurred spontaneously *in vivo*. 3/ Age ranges were quite different: P0-4 in the dish versus P21 and P41 in the Catwalk system. Further experiments should be performed to identify the possible reasons for this apparent inconsistency. 

The group prenatally treated with FSAE also showed a decreased relative paw placement compared to control group. These findings reflect temporal (paw-floor contact) as well as spatial (paw pressure; shift in the ipsilateral paw position) changes in gait. However, the mechanisms underlying the alterations of sensori-motor development are unknown. Similar changes have been described in PCPA treated animals [54]. It is conceivable that prenatal FSAE exposure affects the monoaminergic descending systems during the critical period of neural network formation. 

Taken together, we show that maternal exposure to FSAE alters the excitability of the spinal locomotor network early during development. These alterations are likely associated with significant changes in motor performance and gait in adults. Our results highlight the risk of fenugreek seeds consumption during pregnancy. 
